# Identification of a potent dual-function inhibitor for hIMPDH isoforms by computer-aided drug discovery approaches

**DOI:** 10.3389/fphar.2022.977568

**Published:** 2022-10-26

**Authors:** Meysam Yazdani, Javad Zamani, Seyed Safa-Ali Fatemi

**Affiliations:** ^1^ Department of Systems Biotechnology, Institute of Industrial and Environmental Biotechnology, National Institute of Genetic Engineering and Biotechnology (NIGEB), Tehran, Iran; ^2^ Department of Plant Molecular Biotechnology, Institute of Agricultural Biotechnology, National Institute of Genetic Engineering and Biotechnology (NIGEB), Tehran, Iran

**Keywords:** drug discovery, hIMPDH, immunosuppressant, virtual screening, transplant rejection

## Abstract

Inosine monophosphate dehydrogenase (IMPDH) is a key enzyme in *de novo* biosynthesis of purine nucleotides. Due to this important role, it is a great target to drug discovery for a wide range of activities, especially immunosuppressant in heart and kidney transplantation. Both human IMPDH isoforms are expressed in stimulated lymphocytes. In addition to the side effects of existing drugs, previous studies have mainly focused on the type II isoform. In this study, virtual screening and computer-aided approaches were employed to identify potential drugs with simultaneous inhibitory effects on both human IMPDH isoforms. After Re-docking, Double-step docking, and identification of virtual hits based on the PLANTS scoring function, drug-likeness and ADME-Tox assessments of the topmost ligands were performed. Following further evaluation, the best ligand was selected and, in complex with both isoforms, simulated in monomeric and tetrameric forms using molecular dynamics to evaluate its stability and binding pattern. The results showed a potential drug candidate [(S)-N-(3-hydroxy-1-(4-hydroxyphenyl) propyl)-2-(3-methyl-2,4-dioxo-3,4-dihydropyrimidin-1(2H)-yl) acetamide] with a high inhibitory effect on the two human IMPDH isoforms. This drug-like inhibitor could potentially serve as an immunosuppressant to prevent transplant rejection response by inhibiting B- and T-lymphocyte proliferation. In addition, its effect can be evaluated in various therapeutic targets in which IMPDH is known as a therapeutic target, especially in Covid-19 patients.

## Graphical Abstract

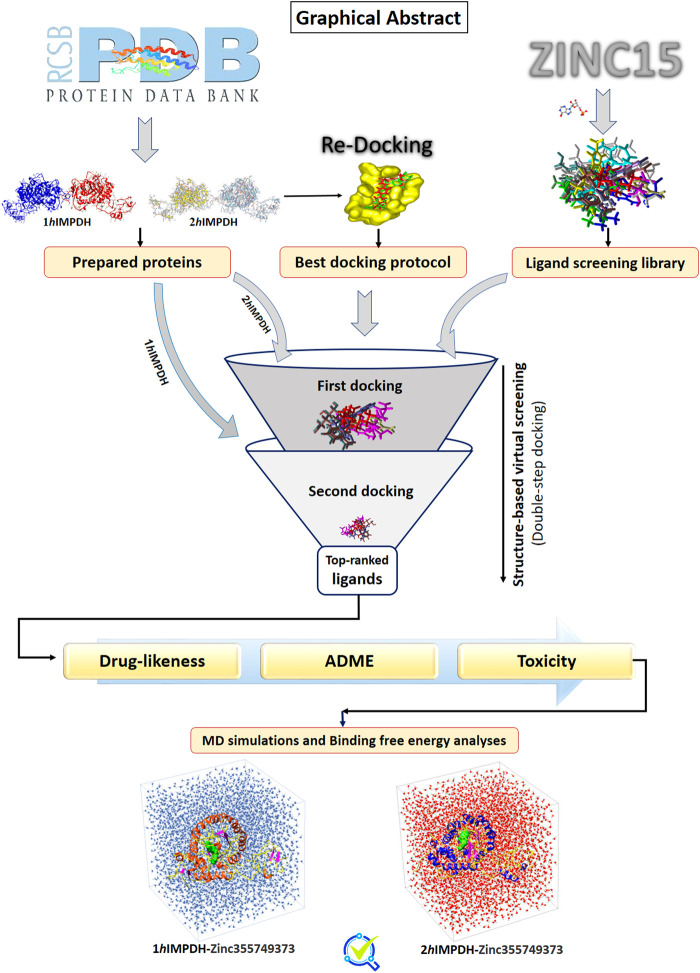

## Introduction

Inosine monophosphate dehydrogenase is one of the most important therapeutic targets in recent years and has been used in the discovery of antiviral (Dunham et al., 2018), antibacterial (Juvale et al., 2019), antiangiogenic (Naffouje et al., 2019), and immunosuppressive (Glander et al., 2021) drugs. IMPDH catalyzes inosine monophosphate to xanthosine monophosphate in the presence of nicotinamide adenine dinucleotide (NAD), and is the rate-determining enzyme in *de novo* guanine nucleotide biosynthesis (Hedstrom, 2009). Humans have two IMPDH isoforms: I and II (Natsumeda et al., 1990). These enzymes are expressed in different ratios in most tissues and cells. The type I isoform is highly expressed in peripheral blood mononuclear cells and expressed at low levels in the thymus. Whereas IMPDH type II is least expressed in the spleen and peripheral blood mononuclear cells (Jain et al., 2004). Both isoforms are significantly expressed in stimulated human lymphocytes (Dayton et al., 1994; Senda and Natsumeda, 1994). Each isoform will gain more importance based on its therapeutic applications.

The two isoforms have approximately 84% sequence identity and 92% similarity in kinetic properties such as substrate affinities, catalytic activities, and Ki values, as well as contains 514 residues (Konno et al., 1991; Saunders and Raybuck, 2000). These enzymes are usually homotetrameric and are stable in this state. Each IMPDH monomer consists of two domains: the catalytic and cystathionine beta-synthase (CBS). The catalytic domain is a (β/α) 8 barrel and harbors an active-site loop located at the end of the β-sheet C-terminal. The most important amino acid in this loop is catalytic cysteine 331 (Cys331), which interacts along other amino acids with IMP, and among IMPDHs is highly conserved (Sintchak et al., 1996; Hedstrom, 2009; Cuny et al., 2017). The CBS subdomain, also known as Bateman domain, appears to play a role in the binding of IMPDH to DNA and suggested by mediating interactions have a function in translation regulation (McLEAN et al., 2004; Mortimer et al., 2008). CBS domains can bind to adenosine derivatives, regulate the activity of proteins and also act as internal inhibitors (Anashkin et al., 2017).

IMPDH inhibitors based on their activities are divided into three groups. The first and second groups occupy the binding positions of IMP and NAD sites, respectively. Finally, the third group ligands binds to allosteric-site that is far from the IMP and NAD pockets (Shu and Nair, 2008). The most important IMPDH inhibitors are Mycophenolic acid (MPA), Mizoribine, Ribavirin (RBV) and Tiazofurin adenine dinucleotide (TAD). All of these drugs suppress the human immune system and exhibit a wide range of antiviral activities. For example, RBV approved for the treatment of infections caused by hepatitis C virus and TAD is the active metabolite of Tiazofurin that is an anticancer and it also has antiviral activity (Ishikawa, 1999; Herrmann et al., 2003; Pankiewicz et al., 2004; Leyssen et al., 2005).

MPA is a potent immunosuppressive drug that inhibits the division and proliferation of B-and T-lymphocytes. This natural product has been approved by the FDA for the prevention of acute rejection of heart and kidney transplantation (Kobashigawa et al., 1998; Johnson et al., 1999). MPA is mostly an uncompetitive inhibitor of both IMP and NAD, and is sometimes considered a non-competitive inhibitor at low NAD concentrations (Allison and Eugui, 1996; Link and Straub, 1996; Gan et al., 2002). Despite the application of MPA, it is easily converted to MPA-7-O-glucuronide, which reduces its efficacy, and also its side effects have been reported (Franklin et al., 1995; Davies et al., 2007).

*In silico* methods have been developed to the investigation and identification of novel drugs (Yazdani et al., 2021). Computational screening of chemical libraries to identify small molecules that bind to a target such as an enzyme or protein receptor, known as virtual screening (Shoichet, 2004; Rester, 2008). Molecular dynamics (MD) simulation methods can be applied at each stage of drug discovery and have a variety of applications (Durrant and McCammon, 2011). After screening, MD widely used to confirm and refine the docking solutions (Salsbury Jr, 2010; Lin, 2011).

In addition to the constraints of existing inhibitors, most of inhibitor development plans have focused on one of the human IMPDH isoforms (Hedstrom, 2009). Considering the great similarity and identity between these two isoforms and their expression in stimulated lymphocytes, a docking-based virtual screening protocol was conducted to introduce a new dual-function ligand that inhibits both IMPDH isoforms.

## Materials and methods

### Preparation of the desired proteins

By the end of 2021, 18 X-ray crystallographic structures have been reported for the IMPDH isoform type II, whereas only one has been reported for type I. Three-dimensional (3D) structures of the IMPDH isoforms; 1NF7 (type II) and 1JCN (type I) at 2.65 and 2.50 Å resolution, were retrieved respectively from the RCSB Protein Data Bank (RCSB PDB) (Risal et al., 2003; Risel et al., 2004). Both structures have two protomers (chains A and B). Because the missing residues at two chains of each structure were the same and equal, chains A were selected as two structures representative. All additional cofactors and co-crystallized ligands in the structures were removed.

### Re-docking

Molegro Virtual Docker (MVD) version 6.0 includes four search algorithms and four scoring functions, that from their combination, various docking protocols it will be obtained (Thomsen and Christensen, 2006). Search algorithms are used to detect ligand orientations into the related conformational space (poses) and to assess and rate these poses to choose the best pose, the scoring function has been applied (Leach, 2001). In this research, two search algorithms, MolDock Optimizer and MolDock Simplex Evolution (MolDock SE), with two scoring functions, PLANTS score and PLANTS score Grid, were used. The accuracy of these protocols was evaluated by re-docking to enhance the success of the molecular docking procedure. To obtain the crystallized ligand position among the four created protocols and select the best protocol, re-docking was performed. For each docking protocol, 1,000 poses were generated, and the lowest score in each protocol was considered as the best pose. The best poses of docking simulation protocols with the co-crystallized ligand position were compared using root mean square deviation (RMSD). Finally, RMSD was calculated by UCSF Chimera (Pettersen et al., 2004), and the lowest RMSD was recognized as the best and most reliable protocol. All re-docking processes were performed using the X-ray crystallographic structure of type II human IMPDH in complex with the RVB ligand (1NF7).

### Ligands screening library preparation

The ZINC 15 database (http://zinc15.docking.org) which is encompasses more than 120 million compounds, including drugs, natural products, metabolites, and annotated compounds was used to select ligands for virtual screening (Sterling and Irwin, 2015). The IMP ligand was used as a reference for the initial screening of the ligand library reconstruction. Initial screening was performed based on the partition coefficient (logP) and molecular weight (Mwt) of the IMP. Predefined subsets were set to drug-like, and the compounds were filtered according to molecular charge, pH range, and reactivity criteria. Finally, the selected ligands were downloaded in 3D conformations in the mol2 format for virtual high-throughput screening.

### Double-step docking

After preparing the target proteins and screening library, the best docking protocol obtained from re-docking was implemented using MVD. In the first docking, the drug-like candidates were docked to the active site of the type II IMPDH crystallographic structure (1NF7). Subsequently, 10% of the best results based on PLANT score were selected for the next step. Next docking was carried out against active site of the crystal structure of type I IMPDH protein PDB 1JCN. After second docking, top twelve ranked ligands were determined and compared with IMP as the main substrate and MPA as an important inhibitor of IMPDH. The parameter settings for all dockings were set to the default MVD. The scoring function was set to an affinity grid resolution of 0.3 Å. Ten runs were performed for each ligand with a threshold energy of 100.0 kcal/mol for pose generation.

### Drug-likeness and ADME-Tox tests

After re- and double-step docking, ADME (Absorption, Distribution, Metabolism and Excretion) and, bioactivity computational prediction, and toxicity analysis were accomplished. The ADMET predictions is used to understand the pharmacokinetic profiles of the chemical compounds. ADME properties including blood-brain barrier, human intestinal absorption, plasma protein binding (PPB), aqueous solubility, intestinal epithelium cell line biological simulations, and toxicity prediction tests such as the Ames test, carcinogenicity, and rat acute toxicity (LD50) were tested by PreADMET and admetSAR servers. The drug-likeness properties were checked using DruLiTo software and SwissADME tool (Daina et al., 2017). Open Bable GUI tools software was used to obtain all the required formats from available mol2 format.

### MD simulations

MD was used to predict the sustainability and estimate the kinetics and thermodynamics of binding ligand-protein complexes obtained from double-step virtual screening.

MD simulations was carried out using the GROMACS 4.6.5. GROMOS 54A7 was used to create proper topologies. The systems were placed at a distance of 2 nm from the cubic box to the protein surface and solvated using the TIP3P model of water. Na+ or Cl-ions were added to neutralize of the system. After solvation and neutralization, the selected docked complexes were subjected to energy minimization using the steepest descent algorithm in 5,000 steps for each simulation. Equilibration of the systems at a temperature of 300 K and pressure of 1 bar was carried out under the NVT and NPT ensembles. To compute the electrostatic interactions and constraints of the bond lengths, the PME method and LINCS algorithm were used, respectively (Zamani Amirzakaria et al., 2021). Eventually, MD runs were performed separately during 50 ns for two complexes in monomeric form.

To validate the results, two complexes with tetrameric form were also subjected to a 500 ns large-scale MD simulation. In addition, the MM-PBSA method (Kumari et al., 2014) was used to evaluate the MD trajectory data in order to calculate the binding free energies of the ligand-receptor complexes.

## Results

### Re-docking

**FIGURE 1:**
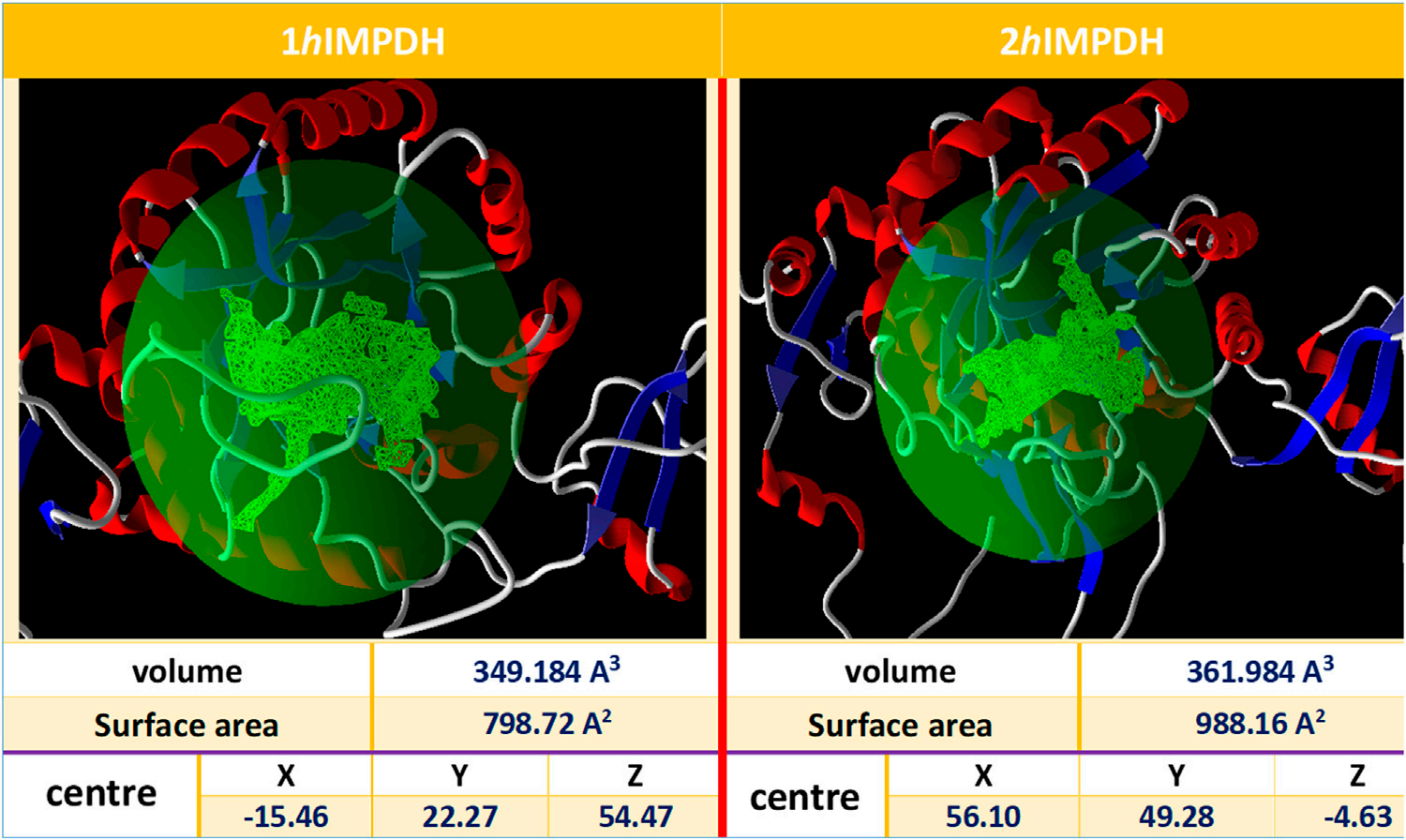
Overview of the selected cavities in both isoforms along with their characteristics.

The IMP sites of the proteins were identified using MVD program (Figure 1). The main residues at this site were as follows: Ser68, Pro69, Met70, Asp71, Thr72, Val73, Thr74, Asp274, Ser276, Gln277, Asn303, Val304, Arg322, Val323, Gly326, Ser327 (2hIMPDH), Cys327 (1hIMPDH), Gly328, Ser329, Ile330, Cys331, Ile332, Thr333, Gln334, Glu335, Val336, Met337, Asp364, Gly365, Gly366, Ile367, Gln368, Met385, Met386, Gly387, Ser388, Leu389, Leu390, Tyr411, and Arg412, Met414, Gly415. These residues are relatively conserved among IMPDH enzymes of different species (Nair and Shu, 2007).

**FIGURE 2:**
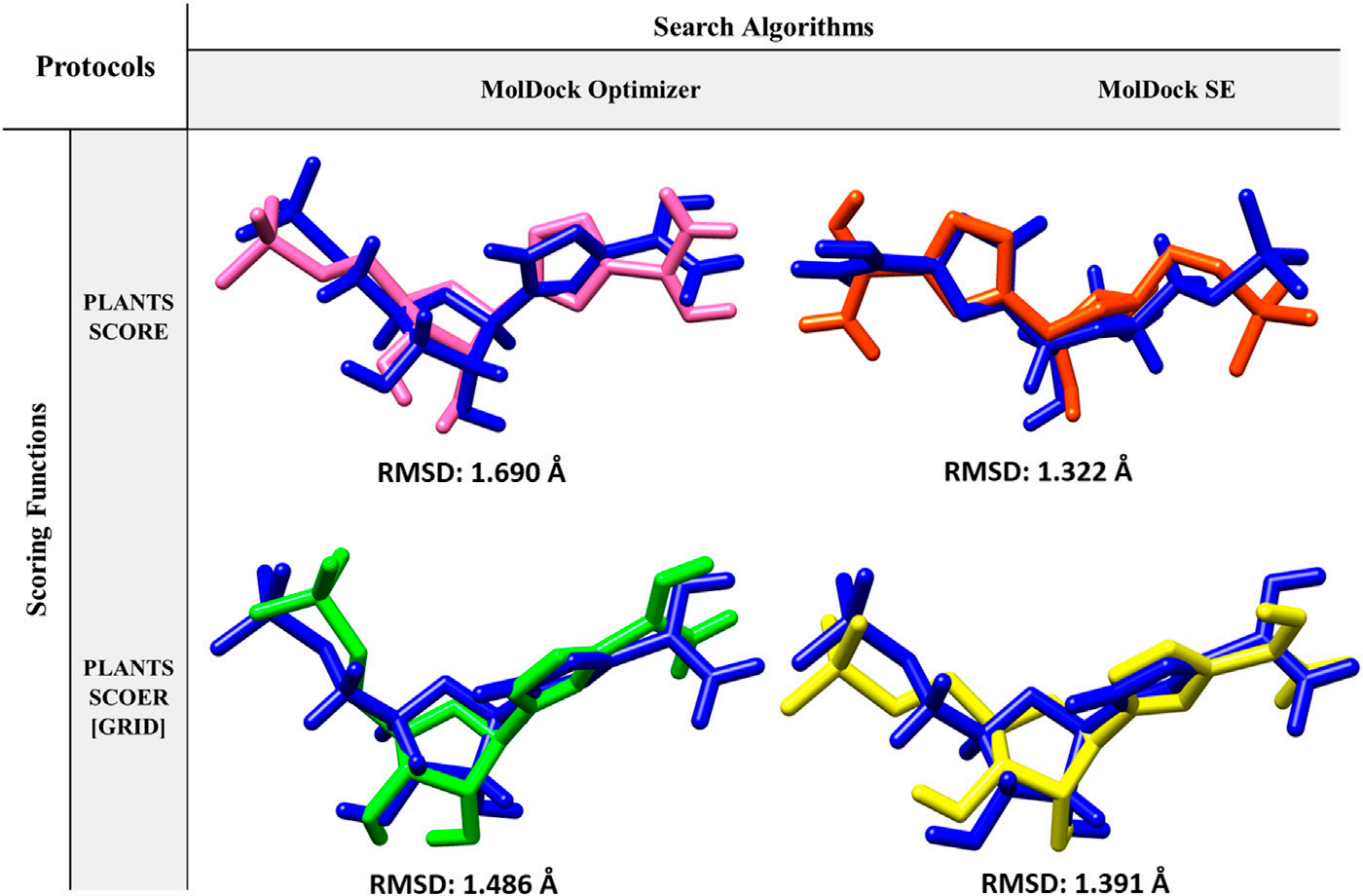
Protocols used in Re-docking to select the best search algorithm and scoring function for virtual screening.

Re-docking is a docking validation procedure that was used to determine which molecular docking algorithms can better predict the co-crystallized ligand position. Based on re-docking results, the best docking protocol was determined based on RMSD results (Figure 2). Comparing the position of the docked ligand with the four mentioned protocols against the co-crystallized ligand position, the MolDock SE search algorithm with the PLANTS SCORE scoring function protocol showed the lowest RMSD (Figure 2). Therefore, choosing this docking protocol appears to be more logical and reliable.

### Double-step docking

A ligand screening library was constructed by applying certain parameters among millions of compounds. Initially, these ligands were docked to the type II hIMPDH isoform. The best results of the first docking stage were considered as the screening libraries for the second docking stage. This step was performed against the type I isoform of this enzyme and with the presence of the top ten percent of the first step docking results. The top ligands in terms of binding energy were determined based on the PLANTS scoring function during double-step docking (Table 1). These ligands have high affinity to both hIMPDH isoforms and can be potential inhibitors. Among these compounds, Zinc355749373 showed a higher affinity for both isoforms than the other ligands. The physicochemical characteristics of these ligands are given in Table 2. According to the initial screening for the construction of the ligand library from the Zinc database, all the ligands were subjected to Lipinski’s rule of five (Ro5). This rule examines five important physicochemical parameters of a compound to assess its pharmacological ability, which leads to filtration of low-absorption ligands (Lipinski et al., 1997). In accordance with Ro5, all the top 12 selected ligands in terms of binding energy had a molecular weight of less than 500 Da, hydrogen bond donors and acceptors were less than 5 and 10, respectively, and their logP did not exceed 5.

**TABLE 1:** Top ranked ligands based on binding affinity against both human IMPDH isoforms.

SN	Ligands	1hIMPDH			2hIMPDH		
		Plant score	Rerank score	HBond	Plant score	Rerank score	HBond
1	Zinc000355749373	−82.5676	−99.253	−5.43796	−82.0586	−111.981	−21.1384
2	Zinc000275637796	−80.8324	−101.283	−9.09656	−77.5632	−94.3062	−13.6505
3	Zinc000361009822	−80.4871	−104.455	−7.97591	−76.1787	−120.019	−18.1332
4	Zinc000354495307	−79.1711	−113.165	−3.96413	−76.2036	−62.1408	−5.60583
5	Zinc000362649164	−77.3334	−106.064	−4.46735	−77.5076	−114.029	−14.0202
6	Zinc000573536990	−76.0579	−99.1823	−8.96631	−78.8602	−114.572	−23.5771
7	Zinc000495649702	−78.66	−107.791	−5.3116	−79.3803	−105.042	−10.9105
8	Zinc000585286331	−77.7542	−82.0037	−3.36807	−82.5813	−106.717	−4.91384
9	Zinc000031937817	−73.3156	−84.7015	−0.93079	−83.8054	−103.981	−12.0494
10	Zinc000217441,397	−77.6534	−90.9367	−8.93364	−82.346	−100.274	−9.72875
11	Zinc000217041949	−73.2131	−80.5838	−5.25843	−83.5722	−52.0103	−8.16447
12	Zinc000048237288	−74.9626	−106.001	−5.07809	−80.4708	−100.163	−7.19021
13	IMP(control)	−60.0844	−81.3601	−7.23357	−59.0203	−80.051	−11.4567

**TABLE 2:** Characterization of topmost ligangds obtained from double-step docking.

Compound	Mol. F	Mol. Wt	logP	HBD	HBA	tPSA	Rot B
Zinc000355749373	C16H19N3O5	333.344	−1.028	3	7	113	6
Zinc000275637796	C16H20N4O5	348.359	−1.069	4	6	127	6
Zinc000361009822	C10H9F3N6O4	334.214	−1.14	2	6	128	4
Zinc000354495307	C17H25N5O3	347.419	−1.397	2	5	112	8
Zinc000362649164	C14H21N7O3	335.368	−1.991	2	8	112	5
Zinc000573536990	C13H16N8O3	332.324	−1.971	1	10	121	3
Zinc000495649702	C15H26N4O5	342.396	−1.134	1	7	85	4
Zinc000585286331	C18H25N3O4	347.415	−1.015	3	5	93	4
Zinc000031937817	C16H28N6O2	336.44	−1.295	2	8	79	6
Zinc000217441,397	C15H19N5O5	349.347	−1.809	3	8	133	4
Zinc000217041949	C16H23N5O4	349.391	−1.027	3	8	116	5
Zinc000048237288	C15H22N6O3	334.38	−1.033	1	8	94	5
IMP(control)	C10H13N4O8P	348.208	−2.152	3	11	185	4

The hydrogen bonds between various atoms of the top ligands and both isoforms are shown in Table 3. The length of the hydrogen bonds formed and the number of these bonds significantly affect the binding energies of the ligands. However, the importance of electrostatic and steric interactions between the ligand and the protein should not be overlooked.

**TABLE 3:** Structures and hydrogen bonds of topmost ligands in complex with IMPDH isoforms over double-step docking.

Ligands	Structure	Hydrogen bond interactions	
		1hIMPDH	2hIMPDH
Zinc000355749373	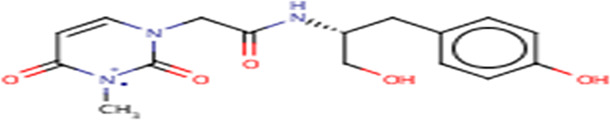	Asp364 … O14 Asn303 … O14 Gly387 … N1 Ser388 … O3	Tyr411 …O24 Ser329 … O24 Ile367 … O3 Asn303 … O14 Asp364 … O14 Asp274 … O20
Zinc000275637796	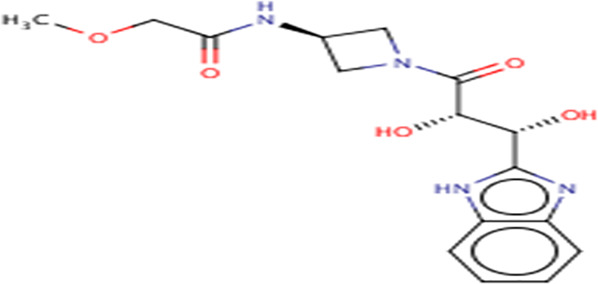	Asn303 … O16 Gly324 … O16 Cys327 … O13 Gly326 … O13 Ser276 … O4 Ser276 … O1	Arg322 … O16 Arg322 … O13 Asp274 … O16 Asp274 … O13 Ser68 … O10
Zinc000361009822	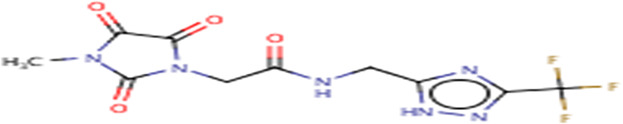	Ser329 … O22 Ser329 … N20 Gly366 … N1 Ile367 … O3	Ser329 … N19 Tyr411 … N19 Asp364 … N10 Ser327 … O5 Gly328 … O5 Cys331 … O3
Zinc000354495307	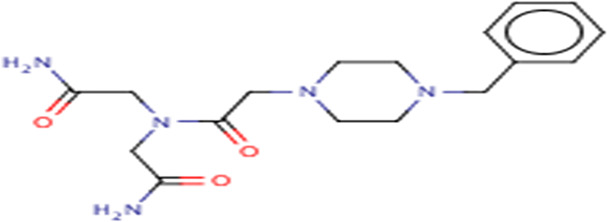	Gln334 … N0 Ser329 … N7 Gly326 … O8	Asp364 … N7 Arg322 … O2 Met414 … O10
Zinc000362649164	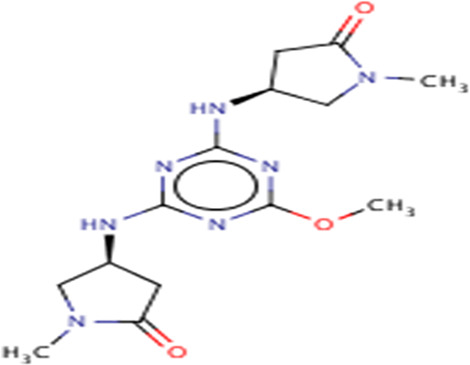	Ser329 … O21 Ser388 … O21 Gly387 … N22 Cys327 … N5 Gly326 … O10	Ser329 … O21 Ser68 … N25 Arg322 … O1 Cys331 … O10 Thr333 … O10
Zinc000573536990	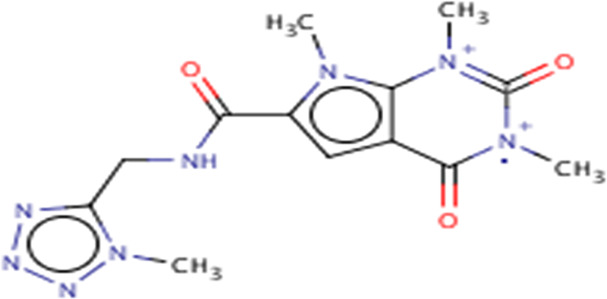	Ser329 … N3 Ser329 … N4 Ser388 … N2 Ser388 … N3 Gly387 … N1 Gly326 … O23	Ser329 … N4 Ser329 … N3 Ser329 … N2 Tyr411 … N2 Ser388 … N2 Gly415 … O23 Thr333 … O19 Cys331 … N16
Zinc000495649702	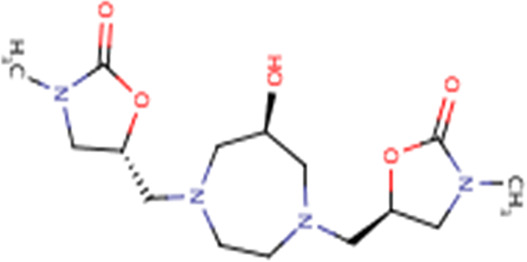	Ser68 … O21 Ser388 … O25 Ser329 … O23	Ser68 … O21 Arg322 … O21 Ser329 … O25
Zinc000585286331	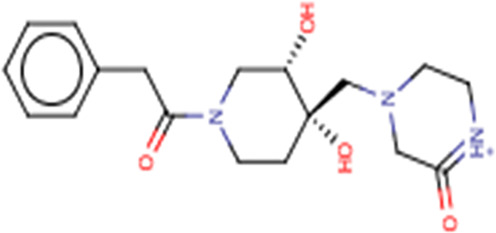	Gly324 … O24 Asn303 … O24	Ser68 … O24 Asp364 … O13 Ile367 … O20
Zinc000031937817	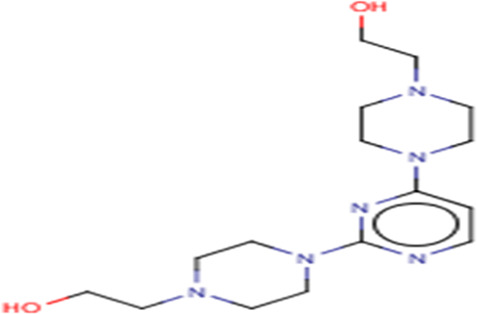	Arg322 … O0	Ser68 … N10 Thr333 … O0 Leu389 … O18 Gly387 … O18 Ser388 … O18 Gln441 … O0
Zinc000048237288	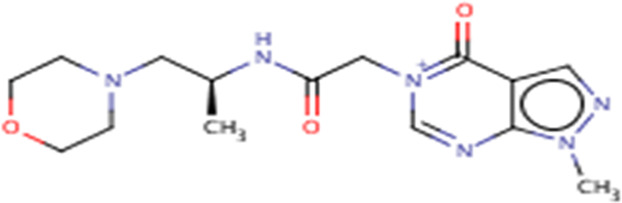	Ser329 … O24 Arg322 … O12 Gly387 … N21 Ser388 … N20	Met414 … O7 Gly415 … O7 Ile367 … N21 Ser329 … O24 Tyr411 … O24
Zinc000217041949	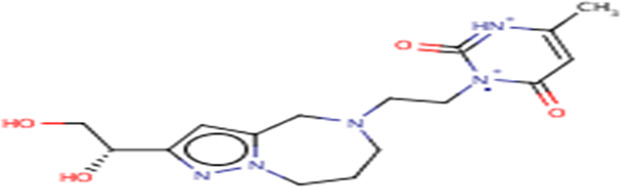	Met337 … O4 Ser276 … O24 Ser329 … O19	Thr333 … O19 Gln441 … O19 Gln441 … O17 Gly365 … O4 Tyr411 … O24 Ser329 … O24
Zinc000217441,397	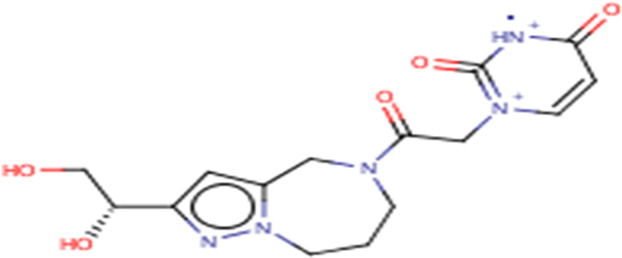	Asp274 … O22 Gly326 … O0 Cys339 … N8 Met337 … O7	Asp364 … O22 Thr333 … O0 Asp274 … N8 Asp274 … O20 Arg322 … O20 Arg322 … O7 Asp364...O22

### Drug-likeness

To choose a ligand as a drug, in addition to having a high affinity for the target, a series of regulations must also be considered. Some of these regulations, such as Lipinsky’s rules, were applied when the ligand library was construct. Filters and other rules such as drug-likeness, ADME and toxicity tests were also reviewed for the top ligands selected from docking. For drug-likeness, Ro5 was investigated as mentioned above, and no violations of this rule were observed for top ligands. Other rules such as BBB Lilkeness, CMC, and MDDR-like rule and filters including Veber (GSK) (Veber et al., 2002), Muegge (Bayer) (Muegge et al., 2001), Ghose (Amgen) (Ghose et al., 1999), and Egan (Pharmacia) (Egan et al., 2000) for top ligands were evaluated. None of the top ligands selected with these considerations showed more than one violation (Table 4), which could be a pleasant result for the selected ligands.

**TABLE 4:** Druglikeness assesments of topmost ligands using DruLiTo and SwissADME tools.

Compounds	Druglikeness							
	Lipinski rule	Ghose filter	CMC like rule	Veber filter	MDDR like rule	BBB lilkeness	Egan filter	Muegge filter
Zinc000355749373	✔	1 violation	1 violation	✔	1 violation	✔	✔	✔
Zinc000275637796	✔	1 violation	1 violation	✔	✔	1 violation	✔	✔
Zinc000361009822	✔	1 violation	1 violation	✔	1 violation	✔	✔	✔
Zinc000354495307	✔	1 violation	1 violation	✔	1 violation	✔	✔	✔
Zinc000362649164	✔	1 violation	1 violation	✔	1 violation	1 violation	✔	✔
Zinc000573536990	✔	1 violation	1 violation	✔	1 violation	1 violation	✔	✔
Zinc000495649702	✔	1 violation	1 violation	✔	1 violation	✔	✔	✔
Zinc000585286331	✔	1 violation	1 violation	✔	1 violation	✔	✔	✔
Zinc000031937817	✔	1 violation	1 violation	✔	✔	1 violation	✔	✔
Zinc000217441,397	✔	1 violation	1 violation	✔	1 violation	1 violation	✔	1 violation
Zinc000217041949	✔	1 violation	1 violation	✔	1 violation	1 violation	✔	✔
Zinc000048237288	✔	1 violation	1 violation	✔	✔	✔	✔	✔

### ADME

To obtain parameters such as BBB, CaCo2, HIA, and CYP of the twelve top ligands, the ADME test was performed. This computational test predicts the absorption, distribution, metabolism, and excretion of compounds, which are very important for the final approval of potential ligands as drugs. To better understand these analyses, IMP as the main substrate and MPA as an approved drug were used as the controls. HIA indicates the intestinal absorption levels in humans. HIA’s high score is important for oral administration of the drug, and compounds with high scores can be easily absorbed by the gastrointestinal tract. Among the top ligands, Zinc573536990, with a full score, showed a high intestinal absorption potential. Most of the compounds with a high probability showed intestinal absorption (Table 5). Ligands with high BBB also indicate high absorption by the blood-brain barrier. This difference in BBB values was due to the different hydrophobicity of the ligands. Caco-2 cells are also a criterion for evaluating cellular interactions, absorption, or transfer from the intestinal epithelial barrier. It was predicted that not all top ligands would cross the Caco-2 cell line. The efflux prediction of pharmacological compounds is done through P-glycoprotein (P-gp) metabolism by the microsomal enzyme family that called cytochrome P450 (CYP450). CYPs are responsible for a large part of drug’s metabolism. It was found that 11 of the 12 top ligands, similar to controls could act as Noninhibitors and Nonsubstrate for CYP450 (Table 5). This means that these ligands cannot disrupt the biotransformation of drug compounds by CYP450 and are not metabolized by this enzyme.

**TABLE 5:** ADME and Toxicity profiles of topmost ligands obtained from PreADMET and admetSAR server.

Compounds	ADME				Toxicity		
	BBB	Caco2	HIA	CYP inhibition/substrate	AMES toxicity	Carcinogens	LD50 in rat
Zinc000355749373	BBB-0.9455	Caco2-0.6193	HIA+ 0.8294	noninhibitor/Nonsubstrate	Non toxic	Non-carcinogens	2.0644
					0.7289	0.7993	
Zinc000275637796	BBB-0.8742	Caco2-0.7464	HIA+ 0.8247	noninhibitor/Nonsubstrate	Non toxic	Non-carcinogens	2.4168
					0.6862	0.8926	
Zinc000361009822	BBB+ 0.8867	Caco2-0.6371	HIA+ 0.9842	noninhibitor/Nonsubstrate	Non toxic	Non-carcinogens	2.4850
					0.5458	0.6374	
Zinc000354495307	BBB+ 0.7883	Caco2-0.6792	HIA+ 0.8793	noninhibitor/Nonsubstrate	Non toxic	Non-carcinogens	2.5121
					0.9017	0.9155	
Zinc000362649164	BBB+ 0.8451	Caco2-0.6141	HIA+ 0.9192	noninhibitor/Nonsubstrate	Non toxic	Non-carcinogens	2.6914
					0.6484	0.8475	
Zinc000573536990	BBB+ 0.9864	Caco2-0.5397	HIA+ 1.0000	noninhibitor/Nonsubstrate	Toxic	Non-carcinogens	2.7100
					0.5329	0.8151	
Zinc000495649702	BBB+ 0.5304	Caco2-0.5644	HIA+ 0.7838	noninhibitor/Nonsubstrate	Non toxic	Non-carcinogens	2.3787
					0.8323	0.9412	
Zinc000585286331	BBB-0.9128	Caco2-0.8411	HIA+ 0.5425	noninhibitor/Nonsubstrate	Non toxic	Non-carcinogens	2.2415
					0.8757	0.9159	
Zinc000031937817	BBB+ 0.5734	Caco2-0.6361	HIA+ 0.9936	inhibitor/Nonsubstrate	Non toxic	Non-carcinogens	2.1940
					0.7675	0.8710	
Zinc000217441,397	BBB-0.6911	Caco2-0.7335	HIA+ 0.9919	noninhibitor/Nonsubstrate	Non toxic	Non-carcinogens	2.0148
					0.5000	0.7951	
Zinc000217041949	BBB-0.8496	Caco2-0.7504	HIA+ 0.9913	noninhibitor/Nonsubstrate	Non toxic	Non-carcinogens	2.0664
					0.5285	0.7823	
Zinc000355749373	BBB-0.9455	Caco2-0.6193	HIA+ 0.8294	noninhibitor/Nonsubstrate	Non toxic	Non-carcinogens	2.0644
					0.7289	0.7993	
IMP (control)	BBB+ 0.8446	Caco2-0.7846	HIA-0.6465	noninhibitor/Nonsubstrate	Non toxic	Non-carcinogens	1.9834
					0.9292	0.9094	
MPA (myfortic) (control)	BBB+ 0.5826	Caco2-0.5583	HIA+ 0.9409	noninhibitor/Nonsubstrate	AMES toxic	Non-carcinogens	2.9907
						0.9619	

### Toxicity

The toxicity of the top ligands was investigated using the following three parameters: AMES, carcinogenesis, and LD50 tests (Table 5). The Ames was used to determine mutagenic ligands. Results revealed that Zinc573536990 is mutagen only. MPA which is used as a control in the Ames test, also showed mutagenic activity. Carcinogenicity analysis did not show any carcinogenic ligands. The higher scores of LD50 for ligands compared to the IMP, revealed that all of them are suitable and non-lethal.

### MD simulations and binding free energy calculations

**FIGURE 3:**
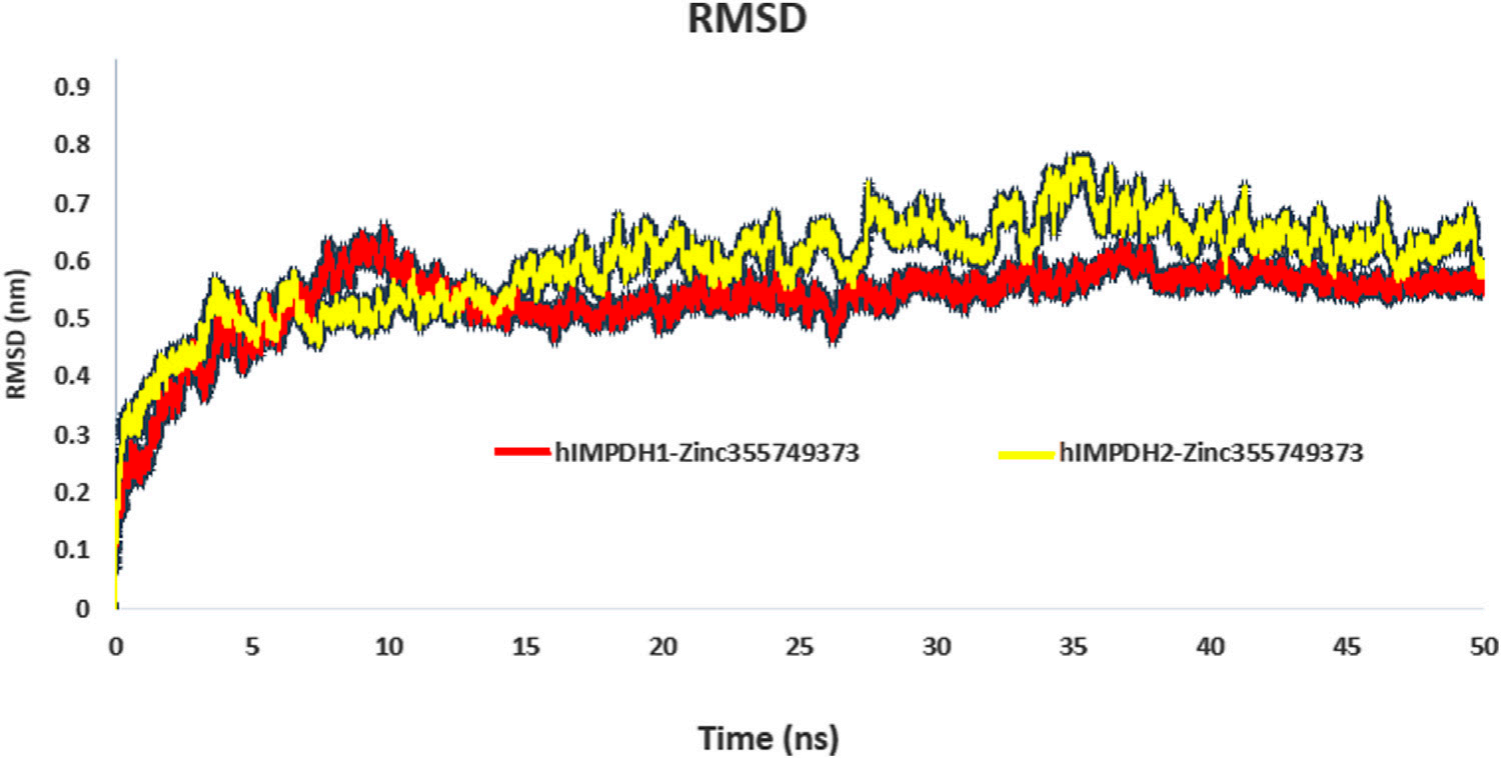
RMSD plot of hIMPDH isoforms (monomeric forms) in complex with selected ligand during the course of 50 ns simulation time.

RMSD profiles obtained from MD simulations over a period of 50 ns were analyzed to evaluate the stability of the ligand-receptor complexes. Figure 3 shows the deviation of the backbone of the initial structure during the simulation period of time. The RMSD values during the simulations of both complexes ranged approximately from 0.07 to 0.75 nm. The RMSD values of both complexes reached to 0.55 nm after 13 ns and no significant fluctuation was observed after that (Figure 3).

**FIGURE 4:**
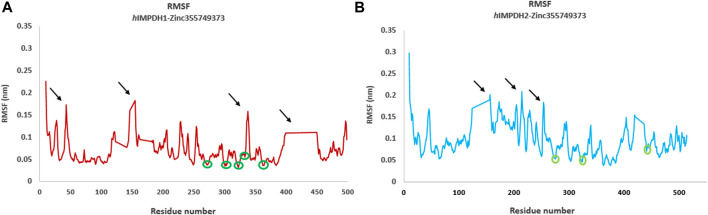
Root mean square fluctuations (RMSF) analysis. RMSF graphs of type 1 **(A)** and type 2 human IMPDH isoforms **(B)** in the presence of best hit ligand over 50 ns MD simulation (monomeric forms). The green circles marked on the diagrams are residues that are hydrogen bonded to the ligand atoms. The areas marked with black arrows that have a high RMSF are the residues that are mostly located in the loop regions.

Root mean square fluctuation (RMSF) plots were used to assess the flexibility and dynamism of the structures. The high peaks marked (Figures 4A,B) in both structures are residues that mainly located in the loop regions. These residues are far from the inhibitor binding site and do not interact with the inhibitor, and their flexibility is expected to not have a significant effect on the stability of the complexes. The areas with lower RMSF values shown in the diagrams, are residues that have a hydrogen bond with the inhibitor and reduced fluctuation.

**FIGURE 5:**
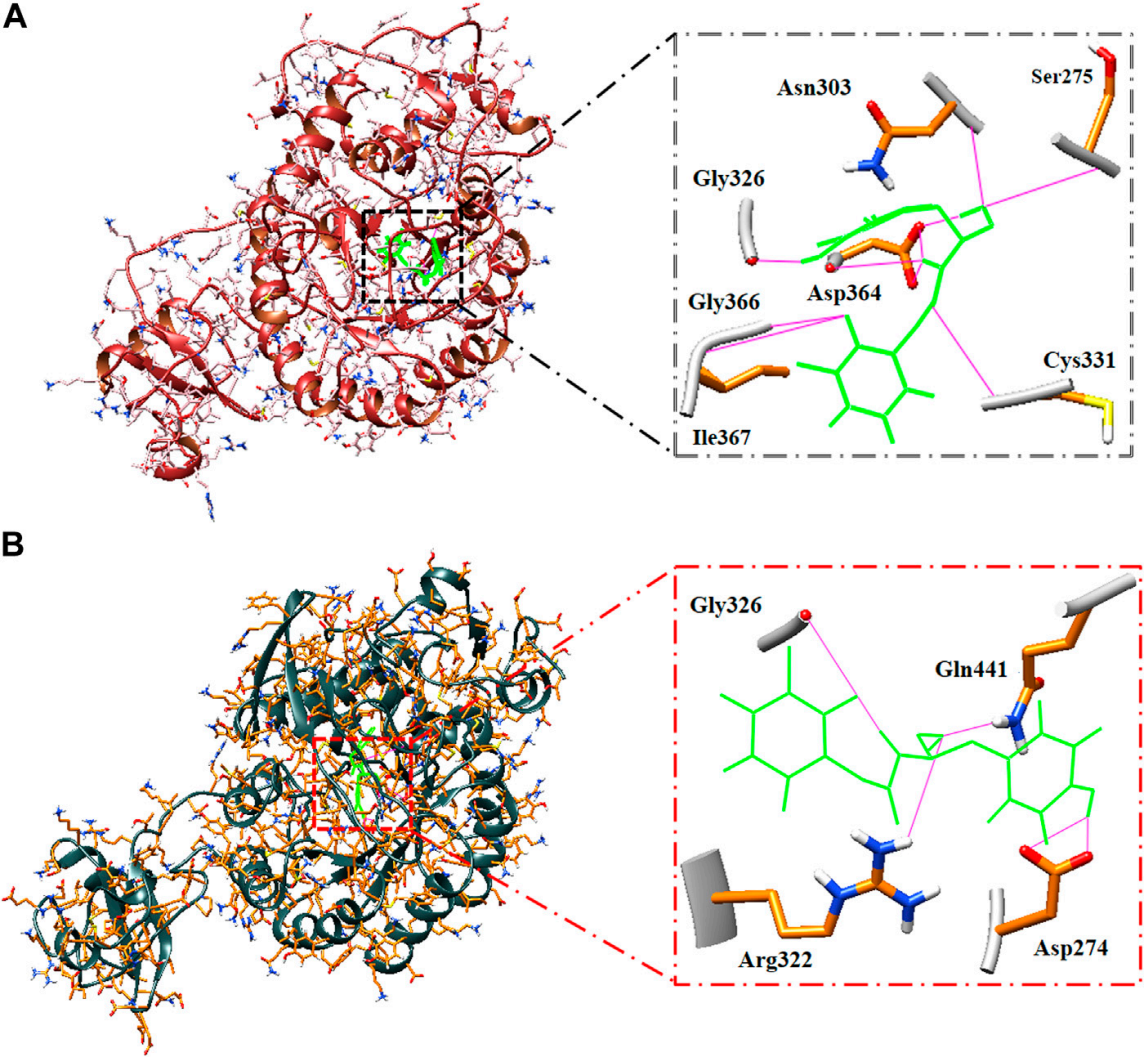
Binding pose and hydrogen bonds of best hit ligand during MD simulation. Type 1 **(A)** and type 2 **(B)** hIMPDH along with its involved residues (highlighted in the figure) in hydrogen bonding in complex with Zinc355749373.

The hydrogen bonds of the best hit ligand with both isoforms during MD simulation are shown in Figure 5. As a result, RMSD and RMSF profiles validated the stability of the inhibitor-proteins complexes and the docking results.

**FIGURE 6:**
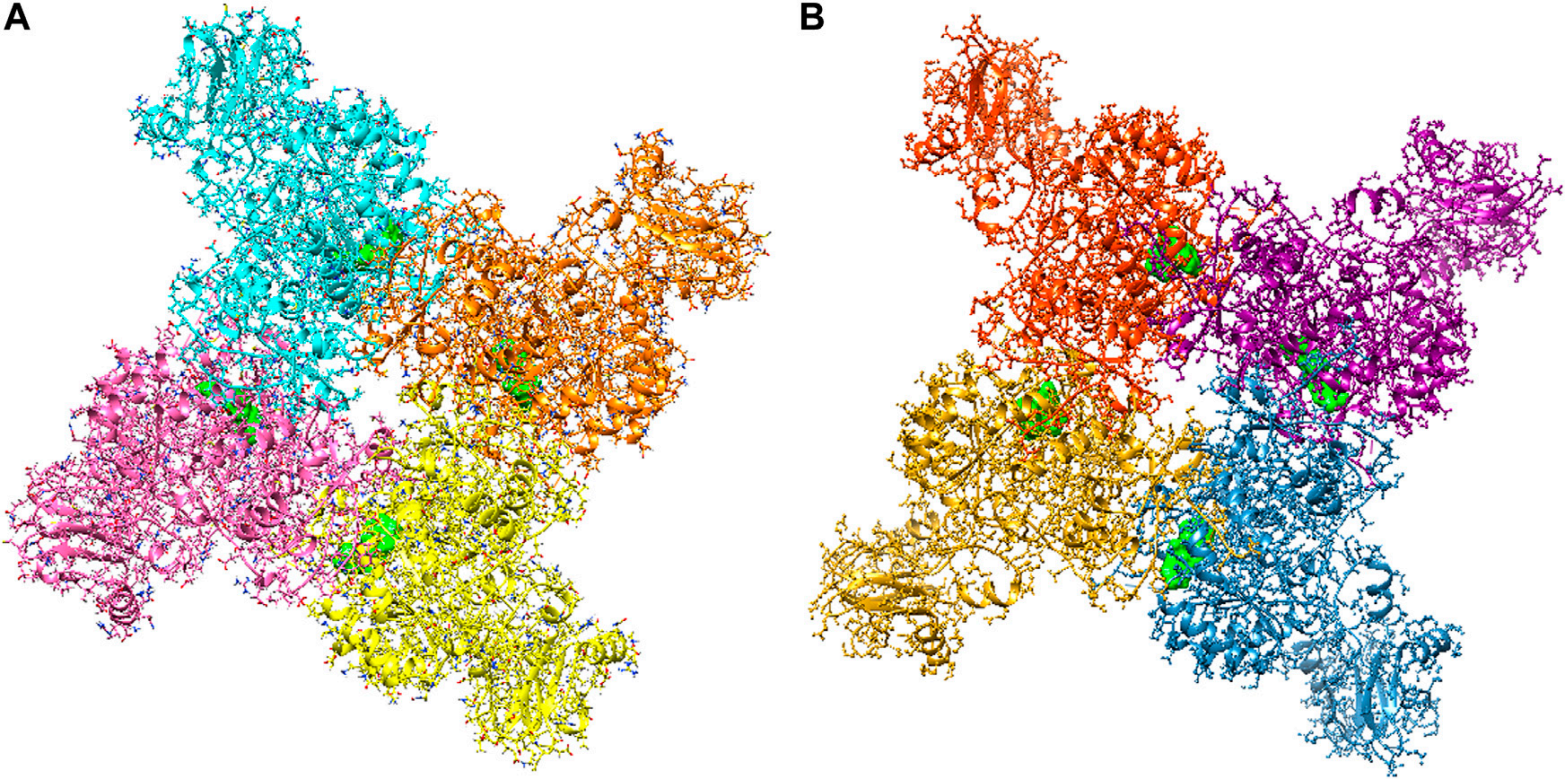
Tetrameric forms of hIMPDH1 **(A)** and hIMPDH2 **(B)** in complex with Zinc355749373 ligand (solid green). The IMP site of each monomer individually interacts with this inhibitor.

**FIGURE 7:**
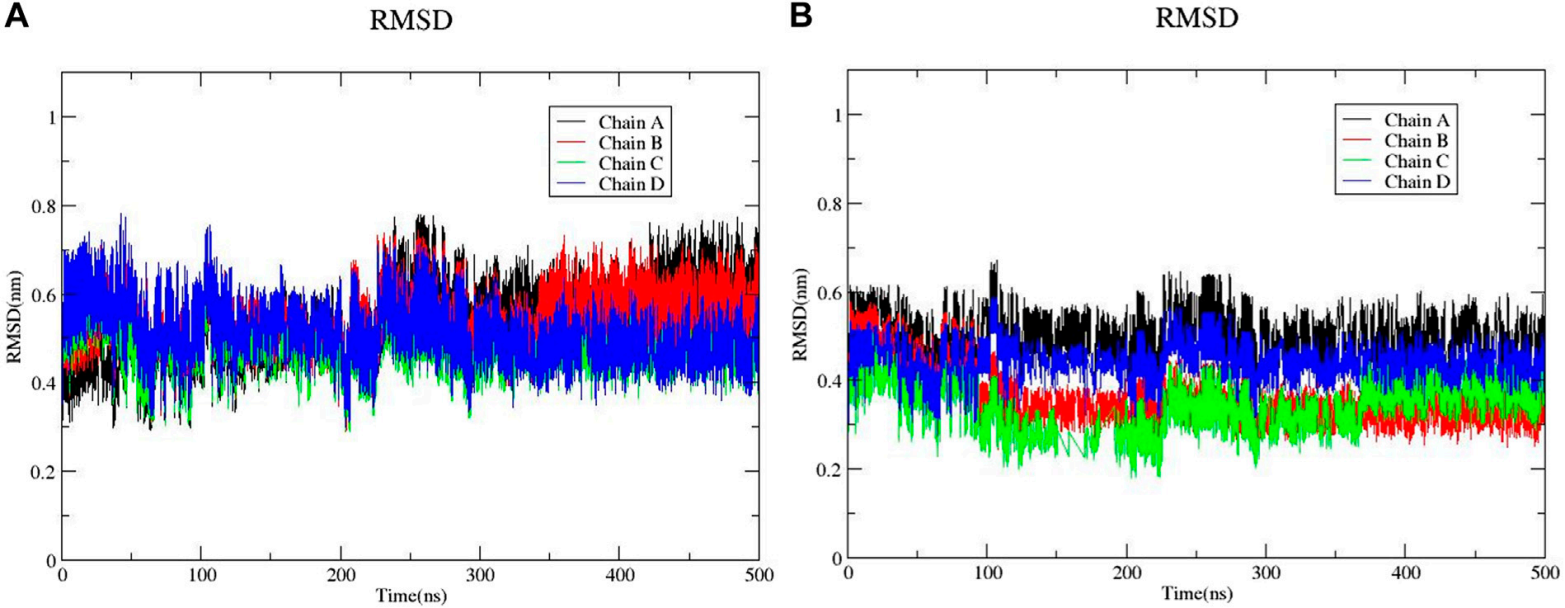
RMSD plots of 1hIMPDH-Zinc355749373 **(A)** and 2hIMPDH-Zinc355749373 **(B)** tetrameric complexes during the course of 500 ns simulation time.

The large-scale MD simulations for tetrameric state which is the functional form of IMPDH isoforms, were performed to validate the monomeric state with more accurate results. The tetrameric forms of 1hIMPDH-Zinc355749373 and 2hIMPDH-Zinc355749373 complexes (Figure 6) were generated using the best docking protocol identified in the re-docking. The RMSD values of tetrameric forms during 500 ns simulations, validated the results obtained from simulations of the monomeric forms (Figure 7). The two complexes in tetrameric form stabilized after 100 ns, and this state continued until the end of the simulations. Given that the proteins are homotetramers, no significant differences were observed in the chains simulation results.

**FIGURE 8:**
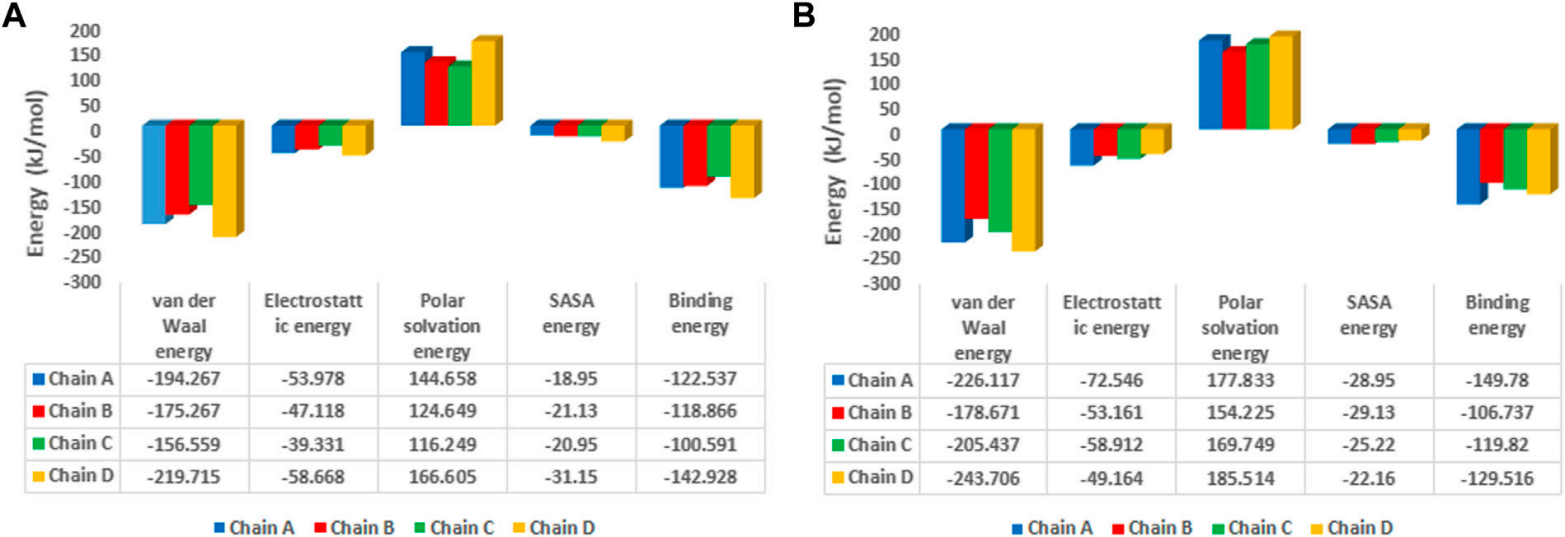
MM/PBSA free energies of 1hIMPDH-Zinc355749373 **(A)** and 2hIMPDH-Zinc355749373 **(B)** complexes in tetrameric form calculated from molecular dynamics trajectory data.

The binding free energy analysis of 1hIMPDH-Zinc355749373 and 2hIMPDH-Zinc355749373 complexes was calculated using g_mmpbsa tool. The binding free energy values and related energies, such as electrostatic interactions, van der Waals forces, polar solvation, and SASA energies for each chain, were obtained by the MM-PBSA method (Figure 8). The mean binding free energies of 1hIMPDH-Zinc355749373 (−121.23 kJ/mol) and 2hIMPDH-Zinc355749373 (−126.46 kJ/mol) tetrameric complexes were in accordance with the docking scores (Table 1) and indicated a high and almost equal affinity of the inhibitor to both isoforms.

## Discussion

### hIMPDH inhibition

The *de novo* biosynthesis of guanine nucleotides has a particular importance for stimulated cell proliferation, because the salvage pathway alone may not be sufficient (Cuny et al., 2017). IMPDH is a rate-determining enzyme in *de novo* guanine nucleotide biosynthesis (Hedstrom, 2009). For this reasons, IMPDH is a potential therapeutic target for a range of diseases including organ transplant rejection, cancer, and viral infections. The two human IMPDH isoforms have different expression levels in different cells of the body. Despite various reports, the mRNA expression of both isoforms increases when lymphocytes and immune responses are stimulated (Dayton et al., 1994). Therefore, inhibition of both isoforms is important for suppressing the immune system. Our *in silico* studies have shown that the Zinc355749373 ligand could potentially inhibit both hIMPDH isoforms and is a potential drug candidate for a variety of purposes, especially to suppress the immune system.

Each known drug or inhibitor of IMPDH acts through a different mechanism. The Zinc355749373 inhibitor identified in this study could act as a competitive inhibitor due to screening among ligands similar to IMP (main substrate). Comparison of the binding energy between two ligands display Zinc355749373 has a higher affinity for both isoforms than the IMP. Zinc355749373 competes with the IMP, binds with a higher affinity to IMPDH, occupies IMP positions, and finally inhibits enzyme activity. This type of binding is reversible and the main substrate can replace the nhibitor at higher concentrations. Based on the available information from zinc 15 database, no activity has been reported for this ligand thus far, and it seems to be a good alternative to MPA. To confirm the results of this research, it is necessary to evaluate this inhibitor *in vitro* and *in vivo* studies. Table 6 lists the several human IMPDH inhibitors.

**TABLE 6:** Some of the most important human IMPDH inhibitors.

Name	Mechanism	Condition	Disesese	Reference
Mycophenolate mofetil	Noncompetitive inhibitor	Approved	Organ transplant rejection	(Wang et al. (2020); Hedstrom, (2009); Shu and Nair, (2008))
	Uncompetitive inhibitor			
	IMP and NAD + site			
Merimepodib	Noncompetitive inhibitor	Phase 2	Hepatitis C virus infection	Wang et al. (2020)
		Phase 2	COVID-19	
Mercaptopurine	—	Approved	Acute lymphoblastic leukaemia	Wang et al. (2020)
Ribavirin	Competitive inhibitor IMP-site	Approved	Hepatitis C virus infection	(Wang et al. (2020); Hedstrom, (2009); Shu and Nair, (2008))
		Approved	Liver Transplantation
Thioguanine	—	Approved	Acute myeloid leukaemia	Wang et al. (2020)
Mizoribine	Competitive inhibitor IMP-site	Approved	Rheumatoid Arthritis	(Hedstrom, (2009); Shu and Nair, (2008))
		Phase 4	Renal Transplant Recipient Patients	
Tiazofurin	Noncompetitive inhibitor NAD-Site Inhibitors	Investigational	Antiviral effects Cancer treatment	(Hedstrom, (2009); Shu and Nair, (2008))
Benzamide Riboside	NAD-Site Inhibitors	Investigational	Angiogenesis Inhibitor	(Hedstrom, (2009); Shu and Nair, (2008))
EICAR	Competitive inhibitor IMP-site	Investigational	Antileukemic and antiviral activity	(Hedstrom, (2009); Shu and Nair, (2008))
Zinc355749373	IMP-site inhibitor	Investigational (*in silico*)	Transplant organs patients and other diseases in which IMPDH is considered as a therapeutic target	This study
	Probably a competitive inhibitor			

### hIMPDH inhibitors as an option to COVID-19 treatment

Increased IMPDH activity in virus-infected cells due to the high need for viral replication in the nucleotide pool highlights the importance of this enzyme as a therapeutic target for viral infections (Nair and Shu, 2007). Therefore, inhibition of IMPDH and reduction of guanine nucleotide levels in infected cells leads to antiproliferative and antiviral effects. Previously, antiviral effects have been reported for some IMPDH inhibitory compounds, such as MPA (Chan et al., 2013), Ribavirin (Koren et al., 2003) and Mizoribine (Saijo et al., 2005) against some members of the coronavirus family, such as SARS-CoV-1 and MERS-CoV. Therefore, IMPDH may be considered as a possible therapeutic target for COVID-19 patients. In a recent study examining the proteome profiling of COVID-19-infected cells, nucleic acid metabolism was identified as one of the metabolic pathways for the major cluster (Bojkova et al., 2020). This finding underscores the limitation COVID-19 proliferation under IMPDH inhibition, which limits the purine biosynthesis. For as much as the replication of coronaviruses depends on the host cellular nucleotide pools. Based on these interpretations, the Zinc355749373 Ligand, which in this bioinformatics study clearly identified the drug potential and its inhibitory effect on both human isoforms of IMPDH, can be evaluated as a potential drug for the treatment of COVID-19 patients. Since the inhibitory effect of Merimepodib, an IMPDH inhibitor, on COVID-19 replication *in vitro* has recently been identified (Bukreyeva et al., 2020).

## Conclusion

This study aimed to identify the potential inhibitors of both human IMPDH isoforms. In addition to side effects and other problems, previous inhibitors generally have a greater inhibitory effect on one isoform. Therefore, an urgent need for newer, safer, and more orally bioavailable IMPDH inhibitors is strongly felt. Furthermore, in patients with acute transplant rejection, inhibition of both isoforms of this enzyme to suppress the immune system can be associated with better results.

The initial results of this study were associated with the introduction of inhibitors of both isoforms in terms of binding energy. Then, by applying various filters and tests, the Zinc355749373 [(S)-N-(3-hydroxy-1-(4-hydroxyphenyl) propyl)-2-(3-methyl-2,4-dioxo-3,4-dihydropyrimidin-1(2H)-yl) acetamide] ligand showed the characteristics of a potential drug ligand. Also, the MD simulation of this ligand in the complex with both isoforms confirmed the docking results. This potential drug inhibitor can be used in clinical assessments for further verification. In addition to evaluating of this dual-function inhibitor as an immunosuppressant, its anticancer and antiviral activities can be appraised *in vitro*, given the current conditions, especially in patients with Covid-19.

## Data Availability

The datasets presented in this study can be found in online repositories. The names of the repository/repositories and accession number(s) can be found in the article/Supplementary Material.
