# Supplementary figures and images for: Identification of a potent dual-function inhibitor for hIMPDH isoforms by computer-aided drug discovery approaches
Source: Front Pharmacol. 2022 Oct 26;13:977568. doi: 10.3389/fphar.2022.977568 (PMC9643795; doi:10.3389/fphar.2022.977568)

# Graphical Abstract

RCSB **PDB**  
PROTEIN DATA BANK

ZINC15

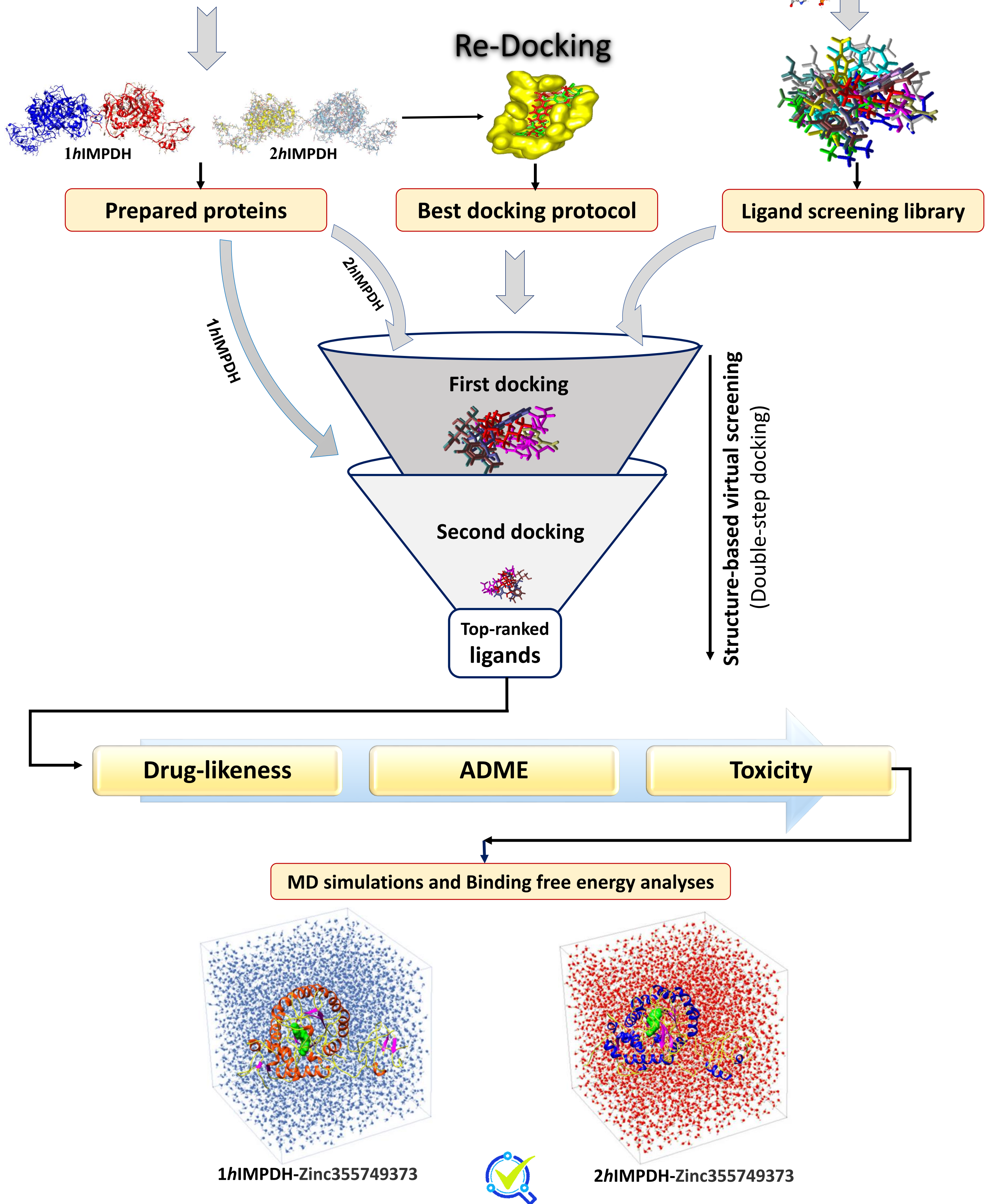

Supplement: Supplementary file 1 [file DataSheet1.pdf]
